# Use of *in Vitro* HTS-Derived Concentration–Response Data as Biological Descriptors Improves the Accuracy of QSAR Models of *in Vivo* Toxicity

**DOI:** 10.1289/ehp.1002476

**Published:** 2010-10-27

**Authors:** Alexander Sedykh, Hao Zhu, Hao Tang, Liying Zhang, Ann Richard, Ivan Rusyn, Alexander Tropsha

**Affiliations:** 1 Laboratory for Molecular Modeling, Division of Medicinal Chemistry and Natural Products and; 2 National Center for Computational Toxicology, U.S. Environmental Protection Agency, Research Triangle Park, North Carolina, USA; 3 Department of Environmental Sciences and Engineering, University of North Carolina–Chapel Hill, Chapel Hill, North Carolina, USA

**Keywords:** acute toxicity, animal testing, computational toxicology, quantitative high-throughput screening, QSAR

## Abstract

**Background:**

Quantitative high-throughput screening (qHTS) assays are increasingly being used to inform chemical hazard identification. Hundreds of chemicals have been tested in dozens of cell lines across extensive concentration ranges by the National Toxicology Program in collaboration with the National Institutes of Health Chemical Genomics Center.

**Objectives:**

Our goal was to test a hypothesis that dose–response data points of the qHTS assays can serve as biological descriptors of assayed chemicals and, when combined with conventional chemical descriptors, improve the accuracy of quantitative structure–activity relationship (QSAR) models applied to prediction of *in vivo* toxicity end points.

**Methods:**

We obtained cell viability qHTS concentration–response data for 1,408 substances assayed in 13 cell lines from PubChem; for a subset of these compounds, rodent acute toxicity half-maximal lethal dose (LD_50_) data were also available. We used the *k* nearest neighbor classification and random forest QSAR methods to model LD_50_ data using chemical descriptors either alone (conventional models) or combined with biological descriptors derived from the concentration–response qHTS data (hybrid models). Critical to our approach was the use of a novel noise-filtering algorithm to treat qHTS data.

**Results:**

Both the external classification accuracy and coverage (i.e., fraction of compounds in the external set that fall within the applicability domain) of the hybrid QSAR models were superior to conventional models.

**Conclusions:**

Concentration–response qHTS data may serve as informative biological descriptors of molecules that, when combined with conventional chemical descriptors, may considerably improve the accuracy and utility of computational approaches for predicting *in vivo* animal toxicity end points.

Traditional research in toxicology relies on animal models to determine adverse effects of chemicals of commercial or environmental importance. From these studies, the mode of action can be suggested for those agents that are deemed hazardous at the molecular or cellular level (Bucher and Portier 2004). One of the most important drawbacks of the current chemical safety testing procedures is that both descriptive and mechanistic toxicology are labor and resource intensive, so only a fraction of the chemicals in commerce and the environment have been evaluated (Andersen and Krewski 2009). Moreover, the recent ban on animal testing of cosmetics in the European Union makes it critical for industry to develop validated alternative approaches (Pauwels and Rogiers 2010). A possible solution to this problem is to develop rapid screening methods based on understanding of toxicity mechanisms and to combine high- information content biology and computational modeling into a predictive framework that can be applied to new chemicals.

High-throughput screening (HTS) assays conducted in multiwell plate format are able to test hundreds to thousands of chemicals for a multitude of biological responses (Houck and Kavlock 2008). As part of the Tox21 collaboration (Collins et al. 2008), the National Institutes of Health Chemical Genomics Center is adapting a large number of quantitative HTS (qHTS) assays to probe biological processes thought to play a role in toxicity of environmental agents.

Accurate prediction of the adverse effects of chemical substances on living systems, identification of possible toxic alerts, and prioritization for animal testing are primary goals of computational toxicology. Progress toward these goals will reduce our reliance on animal testing while ensuring the maximum protection of humans. The prediction of toxicological activity using quantitative structure–activity relationship (QSAR) methods was among the first applications of computational approaches in toxicology. Traditional QSAR models are developed based on chemical descriptors alone (Tropsha 2010). The availability of qHTS concentration–response data offers an intriguing avenue for innovative applications of QSAR modeling in computational toxicology. Indeed, our recent studies have shown that the predictivity of QSAR models for *in vivo* toxicity can be improved when *in vitro* testing data, treated as biological descriptors of chemicals, are combined with traditional chemical descriptors (Zhu et al. 2008, 2009b).

qHTS data allow one to distinguish “active” and “inactive” compounds in individual assays not only based on traditional parameters such as half-maximal effective concentrations (EC_50_) or maximal response but also taking into account the entire range of concentration–response data (Parham et al. 2009). Nevertheless, individual dose–effect points within the concentration–response data have not been previously used as independent parameters in QSAR investigations. In this study, we tested the hypothesis that use of the entire compendium of concentration–response qHTS data (after applying special noise-filtering procedures) can provide novel biological descriptors of chemicals and, when combined with conventional chemical structure descriptors, may improve the accuracy and domain of applicability of computational models predicting *in vivo* animal toxicity [rat half-maximal lethal dose (LD_50_)] of environmental agents. We demonstrate that these hybrid descriptors afford models that are superior to conventional QSAR models in terms of both statistical performance and chemical space coverage. The modeling outputs could also be used to rank *in vitro* assays for utility in predicting toxicity and to suggest optimal chemical concentration ranges for future qHTS experiments.

## Materials and Methods

### Experimental data

#### National Toxicology Program qHTS data

Concentration–response profiles of 1,408 substances screened for their effects on cell viability end points (Inglese et al. 2006; Xia et al. 2008) were available from PubChem (National Center for Biotechnology Information 2008) for 13 cell lines: BJ [human foreskin fibroblast; PubChem BioAssay ID (AID) 421], Jurkat (clone E6-1, human acute T-cell leukemia; AID 426), HEK293 (human embryonic kidney; AID 427), HepG2 (human hepatoma; AID 433), MRC-5 (human lung fibroblast; AID 434), SK-N-SH (human neuroblastoma; AID 435), N2a (mouse neuroblastoma; AID 540), NIH3T3 (mouse embryonic fibroblast; AID 541), HUV-EC-C (human vascular endothelium; AID 542), H-4-II-E (rat hepatoma; AID 543), SH-SY-5Y (human neuroblastoma; AID 544), renal proximal tubule (rat kidney; AID 545), and mesenchymal (human renal glomeruli; AID 546). Each compound was tested at 14 concentrations (0.006–92 μM), and the response was measured as percent change in cell viability compared with vehicle controls using the Cell-Titer-Glo luminescent cell viability assay (Promega, Madison, WI, USA) protocol, which assesses ATP production. The data set was curated as previously described (Zhu et al. 2008): duplicate entries, entries with undefined molecular structure, inorganic, organometallic substances, and mixtures were removed.

#### Rat LD_50_ data

The rat acute toxicity data collection is described in detail elsewhere (Zhu et al. 2009a). There were 7,385 unique organic compounds with rat LD_50_ values expressed as a negative logarithm in units of moles per kilogram.

#### qHTS LD_50_ data set

**Figure 1:**
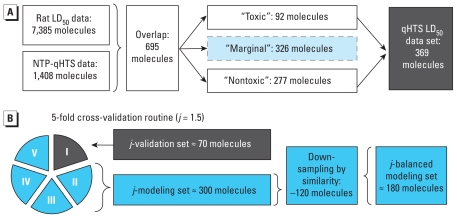
Modeling workflow. (*A*) Preparation of the target data set. (*B*) Modeling procedure for qHTS LD_50_ data set.

For 695 compounds, both qHTS and LD_50_ toxicity data were available (Figure 1A). These were subdivided into three activity categories using the acute toxicity guidelines [Organisation for Economic Co-operation and Development (OECD) 1996; Walum 1998]: 92 “toxic” molecules with −log_10_LD_50_ (mol/kg) > 3, 277 “nontoxic” molecules with −log_10_LD_50_ < 2, and 326 “marginal” molecules with 2 < −log_10_LD_50_ < 3. Only “toxic” and “nontoxic” compounds (*n* = 369) were used for QSAR modeling [see Supplemental Material, Table 1 (doi:10.1289/ehp.1002476)]. Modeled “toxic” compounds correspond to categories 1–3 and “nontoxic” compounds to category 5 of the Globally Harmonized System of Classification and Labelling of Chemicals (United Nations Economic Commission for Europe 2009).

### Molecular descriptors

#### Chemical descriptors

Dragon software (version 5.5; Talete SRL, Milano, Italy) was used to generate descriptors. From the total of 1,911 descriptors, we removed those with low variance (all or all but one value constant) and high correlation (if pairwise *r*^2^ > 0.95, one of the pair, chosen randomly, was removed). The remaining 382 descriptors were range scaled (0 to 1).

#### qHTS-derived descriptors

**Figure 2:**
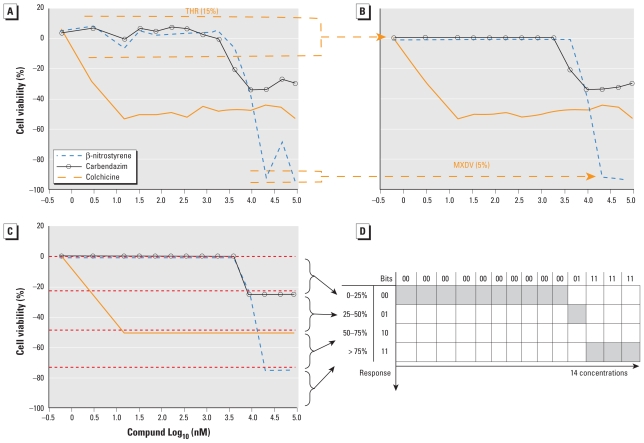
Examples of qHTS concentration–response curves and their noise-filtering transformations. (*A*) Original concentration–response curves for three sample chemicals from the qHTS data set (Jurkat cell line, AID no. 426). (*B*) Data after noise filtering (THR = 15%, MXDV = 5%). THR controls data variation near baseline; MXDV controls deviation from monotonicity. (*C*) Representation of concentration–response by binary fingerprints. (*D*) Concentration–response curve fingerprint of β-nitrostyrene. The *x*-axis indicates the qHTS profile based on 14 concentrations: “00 . . . 00 01 11 11 11” indicates 2^6^ + 2^5^ + 2^4^ + 2^3^ + 2^2^ + 2^1^ + 2^0^ = 127.

First, qHTS profiles were processed by a noise-filtering algorithm developed for this study [see Supplemental Material (doi:10.1289/ehp.1002476)]. Briefly, data points that violated a monotonic concentration–response pattern were replaced by new values calculated from the adjacent data points. The violations of monotonicity were detected by user-defined “baseline threshold” (THR) and “maximum curve deviation” (MXDV) parameters (Figure 2). THR was defined as the largest percent deviation of the response from baseline (i.e., no cell death) within which the response was treated as baseline (Figure 2B), whereas MXDV is the largest percent difference of the response for two adjacent concentration points within which the response is considered unchanged. THR was found to have the greater effect on the outcome of qHTS data processing [see Supplemental Material, Figure 2 (doi:10.1289/ehp.1002476)] and was varied in the studies reported here from 0 (no threshold) to 5%, 15%, and 25% while MXDV was kept constant at 5%. Second, processed qHTS data were used to generate biological descriptors for each compound. Each descriptor type was defined by the concentration/cell line; thus, 14 “concentration–response” biological descriptors for each of the 13 cell lines were generated, for a total of 14 × 13 = 182 descriptors for each set of THR/MXDV. The descriptor value was the modified response measurement. These qHTS-derived descriptors were considered as independent parameters in QSAR models. Third, the modified response value for each dose was converted into a binary “fingerprint” (chosen arbitrarily: “00” if < 25% of maximum response, “01” if 25–50%, “10” if 50–75%, and “11” if > 75%) which may be used to describe the shape of the curve for each compound (Figure 2C,D) but not to interpret the modeling results.

### QSAR modeling

Figure 1B shows the modeling workflow. Key steps of the workflow, to ensure that statistically significant and externally predictive classification models are generated (Tropsha 2010), are described below. The classes being predicted are identical to those in the LD_50_ data set: “toxic” and “nontoxic” according to the acute toxicity guidelines (OECD 1996; Walum 1998).

#### Five-fold external validation

The qHTS LD_50_ data set (consisting of 369 unique organic compounds) was divided, by random selection, into five nearly equal subsets (≈ 70 molecules). Five models were developed independently, whereby 80% of the chemicals were used as a training set and the remaining 20% were used as a test set.

#### Balancing modeling sets

It is well known (Chawla 2005) that an unbalanced (more inactive than active compounds) modeling set usually results in a poor QSAR model. To account for 3:1 dominance of nontoxic compounds, each modeling set (≈ 300 molecules) was subjected to a down-sampling procedure [see Supplemental Material (doi:10.1289/ehp.1002476)] that eliminated a fraction of nontoxic molecules most structurally dissimilar from toxic molecules to achieve approximately a balanced ratio of toxic to nontoxic compounds.

#### Modeling algorithms

Random forest (Breiman 2001) and *k*-nearest neighbors (*k*NN) (Golbraikh et al. 2003; Shen et al. 2002; Zheng and Tropsha 2000) algorithms were used [see Supplemental Material (doi:10.1289/ehp.1002476)]. Each balanced modeling set was subdivided into 20 training/test subsets using the sphere exclusion algorithm (Golbraikh et al. 2003). The predictive power of resulting models was characterized by the correct classification rate (CCR) = 0.5(sensitivity + specificity), where sensitivity (specificity) is the correctly predicted fraction of “toxic” (“nontoxic”) compounds.

#### Applicability domain of *k*NN QSAR Models

Because *k*NN models interpolate activities from the nearest neighbor compounds in the relevant training sets, a special applicability domain (i.e., similarity threshold) should be introduced to avoid classifying compounds that differ substantially from the training set molecules. The detailed description of the applicability domain is available elsewhere (Tropsha 2010).

#### Robustness of QSAR models

*y*-Randomization (randomization of response) is widely used to establish model robustness (Ruecker et al. 2007). The process consists of rebuilding models using randomized activities and then assessing their performance on the external set. This procedure was repeated five times, and the one-tailed *t*-test *p*-value was calculated, which is the probability that a randomized model could achieve a CCR value comparable to that of the best models built with actual activities. If *p* < 0.05, the models are discarded.

## Results and Discussion

### qHTS data improve QSAR model accuracy

The cell viability qHTS assays have been extensively validated and are known to give reproducible results [e.g., half-maximal activity concentration (AC_50_) values] in toxicity screening studies (Inglese et al. 2006; Xia et al. 2008). These data, when converted to binary “biological” descriptors, were shown previously to improve the accuracy of conventional, chemical descriptor-based QSAR models of rodent carcinogenicity (Zhu et al. 2008). The same simple binary descriptors, however, did not improve QSAR models of the acute rodent toxicity (i.e., LD_50_) data set used in this report (data not shown). However, qHTS assays contain full concentration–response information, enabling derivation of multiple “biological” descriptors using a noise-filtering algorithm (Figure 2B).

The initial use of these novel qHTS-derived descriptors alone did not result in robust classification models of rat acute toxicity (data not shown). This observation was similar to those of our previous studies (Zhu et al. 2008) showing that “binary” biological descriptors alone, derived from these same qHTS data, did not correlate well with rodent carcinogenicity. *In vitro* screening, even in as many as 13 cell lines, may not capture the complex biological mechanisms of *in vivo* toxicity.

**Figure 3:**
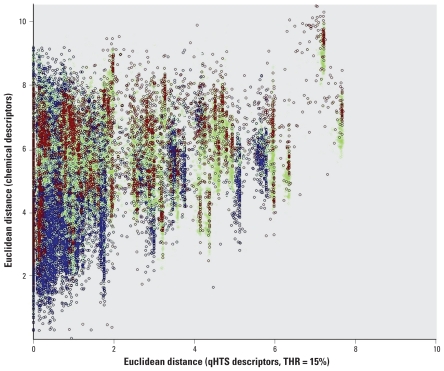
Pairwise Euclidean distances in the chemical (*y*-axis) and biological (*x*-axis) descriptor space for the qHTS LD_50_ data set. Dots represent compound pairs; colors reflect *in vivo* toxicity: blue, pairs of nontoxic compounds; red, pairs of toxic compounds; green, pairs where one compound is toxic and another nontoxic.

We then examined the relationships between the “chemical” and qHTS-derived “biological” descriptors. Following standard cheminformatics procedures, we calculated and plotted pairwise similarities between compounds estimated by respective Euclidean distances using either biological or chemical descriptors (Figure 3). We found no correlation between any two sets of descriptors; that is, chemical similarity is perceived differently by the biological versus chemical descriptors. We conclude from this analysis that both sets of descriptors may bring unique features to models when used simultaneously.

Next, we built QSAR models of acute rat toxicity using chemical descriptors only (Table 1). Based on the external validation set, mean accuracy of the models was > 75%, which supports the utility of chemical descriptor–based QSAR models for the acute rat toxicity end point. To determine whether qHTS-derived “biological” descriptors could improve the model predictivity, we used hybrid, chemical–biological sets of descriptors. When we used unprocessed qHTS descriptors, the model accuracy was dampened (Table 1, THR = 0%), likely due to high noise levels (i.e., random variation) in the concentration–response profiles. However, hybrid models based on the noise-filtered qHTS data showed significantly improved external classification accuracy compared with models based on chemical descriptors alone or hybrid descriptors with untreated qHTS data. Three hybrid models (Table 1, THR = 5%, 15%, and 25%) showed similar performance, indicating that relatively minor correction of the baseline response results in a significant improvement of the model performance. In further analysis, we used the arbitrary value of THR = 15%.

### qHTS data improve QSAR model coverage

**Figure 4:**
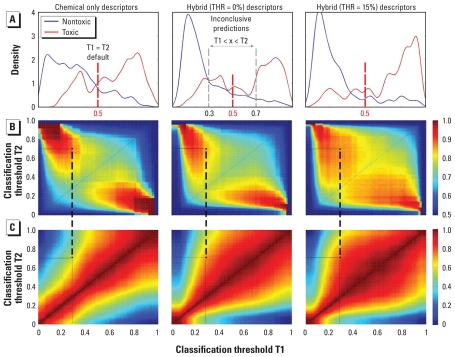
External prediction results of *k*NN models using different classification criteria: distribution of the predicted values (*A*) and heat maps illustrating classification (*B*, CCR) and coverage (*C*, percent chemicals within the applicability domain) results for each pair of classification thresholds T1, T2 (i.e., “nontoxic” < T1 ≤ “not covered” < T2 ≤ “toxic”). Red dashed (*A*) and diagonal (*B*,*C*) lines denote a default single-threshold classification (T1 = T2 = 0.5). Gray (*A*) and black (*B*,*C*) dashed lines denote an example of double-threshold classification (T1 = 0.3 and T2 = 0.7).

We based the classification *k*NN QSAR method in this study on an ensemble of models that uses a consensus scoring scheme whereby an average value of the binary classifications from all individual models (0 = “nontoxic,” 1 = “toxic”), for which a chemical was found within the respective applicability domains, is recorded. The average “prediction” value could fall anywhere within the range between 0 and 1. The results reported in Table 1 are based on a consensus classification using 0.5 as a threshold (i.e., average value > 0.5 is predicted “toxic,” < 0.5 “nontoxic”). However, the *k*NN model’s classification stringency can be adjusted by applying individual thresholds to each class (e.g., ≤ 0.3 is nontoxic, ≥ 0.7 toxic) and treating all inconsistent classifications (e.g., between 0.3 and 0.7) as inconclusive. Although the accuracy of the classification may improve when stringent thresholds are applied, the coverage of the model (i.e., a fraction of the compounds that may be classified because of the applicability domain limitations) is eroded. To explore the relationship between the predictivity and coverage of the models based on chemical or hybrid [original or filtered (15% THR) concentration–response data] descriptors, we have determined the CCR and coverage of the models with varying classification thresholds (Figure 4).

The distribution of the consensus model predictions (Figure 4A) for the test compounds shows that the hybrid descriptor models with noise-filtered qHTS data exhibit most favorable separation of “toxic” and “nontoxic” compounds. Importantly, when CCR (Figure 4B) and coverage (Figure 4C) are plotted as heat maps, it is evident that the hybrid descriptor models with noise- filtered qHTS data have not only high accuracy but also higher coverage at lower thresholds. For example, when fairly strict classification criteria (e.g., ≤ 0.3 for nontoxic, ≥ 0.7 for toxic) are applied, all three types of models can achieve similar classification accuracy (CCR ≈ 86%), yet the coverage is considerably higher for the hybrid models (81% vs. 57%; connected dots in Figure 4C), implying that hybrid models are expected to make accurate predictions for substantially more external chemicals, which is an important model feature for prioritizing new chemicals for *in vivo* testing. Furthermore, the consensus classification value correlates well with LD_50_ [see Supplemental Material, Figure 5 (doi:10.1289/ehp.1002476)].

### Comparative analysis of hybrid QSAR

To evaluate robustness of the classification models, we used the *y*-randomization test (see “Materials and Methods”) applied to the representative hybrid descriptor model with noise-filtered (THR = 15%) qHTS data and the model based on chemical descriptors only. All *y*-randomized models were significantly worse (one-tailed *t*-test *p* < 0.05) than respective real ones, with CCR values < 0.52 in all cases.

We also compared the performance of models developed in this study with that of the widely used commercial toxicity predictor software TOPKAT (Toxicity Prediction by Komputer Assisted Technology) (Venkatapathy et al. 2004). There were 87 molecules present both in our qHTS LD_50_ data set and in the previously reported external validation set (Zhu et al. 2009a) of TOPKAT. Because TOPKAT generates continuous LD_50_ predictions, we made binary classifications using the same criteria as applied in the case of the qHTS LD_50_ data (see “Materials and Methods”); 52 molecules were classified as 11 “toxic” and 41 as “nontoxic” compounds, and the remaining 35 had “marginal” activity (Table 2). Although the hybrid models based on the noise-filtered qHTS data gave CCR values > 0.85, both our chemical descriptor-based models and those of TOPKAT (also based on chemical descriptors only) showed lower predictivity (CCR of 0.75–0.77 or 0.69, respectively; note the dramatic improvement in sensitivity, that is, accuracy in predicting toxic compounds, of our models vs. TOPKAT, 73–91% vs. 43%, respectively, with minor drop in specificity, 83–85% vs. 93%, respectively). These results further support the use of hybrid chemicobiological descriptors in QSAR modeling of chemical toxicity.

### Chemical and biological descriptors are both important for accurate prediction of acute rat toxicity

The QSAR modeling approaches used here allow for the analysis of individual descriptors that appear frequently in models with high classification accuracy. To this end, we further examined the hybrid descriptor-based *k*NN model with noise-filtered (THR = 15%) qHTS data.

**Figure 5:**
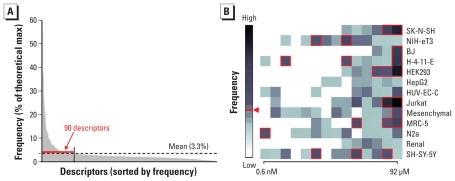
Occurrence frequencies of the descriptors in the hybrid *k*NN (THR = 15%) model (*A*) and relative frequencies of qHTS biological descriptors (*B*). Max, maximum. The fraction of most frequent descriptors selected by mean occurrence is marked by a dashed line (*A*) and by a red arrowhead and red boxes (*B*).

In total, among five splits of the modeling set (Figure 1), we generated > 7,000 individual *k*NN models. Figure 5A shows that, on average, each descriptor appeared in 3.3% of all models. We determined that 90 descriptors had above-average frequency, of which 21 were qHTS-derived descriptors (Figure 5B). The apparent imbalance between chemical and biological descriptors is due to a corresponding imbalance (4:1) in the total number of descriptors of each class used for modeling.

The top descriptor overall, with as high as 61% occurrence, was the Jurkat cell viability response at the highest concentration tested (92 μM). Similar to the observation made in our previous studies (Zhu et al. 2008), the Jurkat cell line was found to be the most significant biological descriptor for predicting *in vivo* toxicity, followed by the SK-N-SH cell line. Jurkat is a human tumor cell line derived from T-cell leukemia, and it grows in suspension with a relatively fast doubling time of about 22 hr. This cell line retains some metabolic capacity toward xenobiotics and is used frequently for *in vitro* testing (Nagai et al. 2002). We found that HepG2 and renal proximal tubule cell lines generated the least informative biological descriptors. Actually, almost all cell lines had model-informative responses over the top six concentrations tested; we derived fewer informative data from the mid to lower part of the concentration range (Figure 5B). Independent of assay hit frequency, however, the modeling success suggests that the modes of action for chemicals that cause overt toxicity *in vivo* may, at least in part, correspond to those operative *in vitro*. Interestingly, the qHTS descriptor representing response at the lowest concentration tested (0.6 nM) in the N2a cell line was indicative of nontoxic classification (of 26 compounds with nonzero response at 0.6 nM, 1 was toxic, 9 were nontoxic, and 16 were marginal). This result underscores the need for including sufficiently high and low concentrations for *in vitro* screening of chemicals.

Table 3 summarizes the most frequently selected chemical descriptors. They fall into several chemical categories consisting of halocarbon compounds, sulfur-containing molecules (mainly thiophosphates), and aromatic structures. These chemical classes are known for their prevalent toxicity (Denison 1990; Vittozzi et al. 2001). Several of the descriptors are likely to serve as secondary features within classes, to afford recognition of specific subclasses of molecules that have either low or high toxicity.

In addition, we argue not only that there is value in better understanding what descriptors were successful at predicting activity class, but also that it is useful to analyze the “classification outliers”—that is, those chemicals that the models failed to predict accurately. Because both chemical structure–based and qHTS profile–based descriptors are available, we can determine whether certain chemical classes of the consistently correctly/incorrectly classified compounds have similar concentration–response curve fingerprints (see “Materials and Methods” and Figure 2D), as well as cases where qHTS results are less reliable or informative to the model success. Table 4 illustrates several sample comparisons using qHTS fingerprints derived from the concentration– response curves in the 13 cell lines. For example, correctly classified polychlorinated phenols, aliphatic alcohols, and acetates (Table 4, items 1–3) exhibit similar *in vitro* concentration–response profiles and *in vivo* toxicity. In contrast, a pair of benzaldehyde molecules (Table 4, item 4) have markedly different qHTS profiles, with one profile indicating more potential toxicity, whereas both are considered inactive *in vivo*; in this case, chemical descriptors perceive the chemicals as similar in relation to toxicity. For alkyl halides and nitriles (Table 4, items 5 and 6), *in vitro* screening failed to detect toxicity, whereas they are positive for *in vivo* toxicity (except for volatile bromoethane and acetonitrile), but in the case of phenylenediamine derivatives and alkyl aldehydes (Table 4, items 7 and 8), the agreement between *in vitro* and *in vivo* results is higher.

For some misclassified compounds (e.g., bromoethane, acetonitrile, or methyl vinyl ketone; Table 4, items 5, 6, and 9), the errors may be related to metabolism. For example, in the case of alkyl nitriles, their toxicity is known to be caused by the hydrogen cyanide metabolite (Willhite and Smith 1981). Other reasons for failure of the model to accurately predict could include certain physical properties (e.g., volatility) and chemical uniqueness, that is, when a “structural outlier” is the only representative of a certain mechanism of toxicity. These factors may help explain incorrect classification of iodoform and methyl isocyanate, which are small volatile molecules with inactive qHTS profiles but are known to be toxic *in vivo*.

These results suggest that a strategy for refining hybrid models could be to tailor their applications based on the success or failure of the global consensus models in local regions of chemical space. For example, in regions of chemical space where pharmacokinetics (e.g., metabolism or absorption) challenges *in vitro*–*in vivo* comparisons, models could be trained to rely exclusively on chemical descriptors, and the generation of qHTS data would be less crucial. In other areas of chemical space, where qHTS results add significantly to model performance, generation of qHTS results would be considered a higher priority, and in these cases, our results show the importance of using both short-term assays and advanced cheminformatics approaches for predicting *in vivo* toxicity assessment.

## Conclusions

We found qHTS *in vitro* data for cell viability alone to be insufficiently accurate classifiers of *in vivo* acute lethal toxicity. Nevertheless, the *in vitro* data, especially concentration– response qHTS profiles, can improve the results of QSAR modeling of *in vivo* end points compared with conventional QSAR models using only chemical structure descriptors. To achieve this outcome, it was essential to apply a novel noise-filtering algorithm to the concentration–response qHTS data. The resulting biological qHTS descriptors afford improved hybrid chemicobiological models over those based on chemical descriptors alone. Importantly, hybrid descriptors from noise-filtered qHTS data also enhanced the model coverage, which is essential for applying models to large and diverse chemical libraries of environmental concern. Obviously, if hybrid models are to be applied for predicting *in vivo* toxicity, *in vitro* screening data are needed, yet the value of qHTS-based modeling for unknown agents may depend strongly on the chemical structure. Specifically, performance of models in local regions of chemical space, as inferred here from feature descriptors included in successful models, could be used to prioritize where qHTS data would be most informative and important for prediction. The results of the present study provide compelling support for increasingly sophisticated and tailored predictive approaches that incorporate all available information (chemical, biological, and concentration–response) in modeling.

## Figures and Tables

**Table 1:** CCRs of 5-fold external validation for *k*NN and random forest models.

Split no.	Chemical descriptors only	Hybrid			
		THR = 0%	THR = 5%	THR = 15%	THR = 25%
*k*NN					

1	0.75	0.74	0.79	0.79	0.79
2	0.76	0.67	0.79	0.79	0.79
3	0.75	0.74	0.90	0.86	0.87
4	0.71	0.79	0.78	0.81	0.74
5	0.83	0.77	0.81	0.82	0.83
Mean	0.76	0.74	0.81*	0.81*	0.80*

Random forest					

1	0.75	0.70	0.79	0.80	0.77
2	0.77	0.79	0.84	0.83	0.82
3	0.80	0.77	0.85	0.88	0.86
4	0.74	0.74	0.71	0.74	0.71
5	0.84	0.83	0.83	0.83	0.83
Mean	0.78	0.77	0.80*	0.82*	0.80*

**Table 2:** Classification results for external validation set.

	TOPKAT	Chemical descriptors only		Hybrid descriptors			
				THR = 0%		THR = 15%	
		*k*NN	RF	*k*NN	RF	*k*NN	RF
CCR	0.69*	0.75	0.77	0.70	0.80	0.88	0.87
Sensitivity	0.45*	0.73	0.73	0.55	0.82	0.91	0.91
Specificity	0.93*	0.78	0.80	0.85	0.78	0.85	0.83

RF, random forest. Each misclassification corresponds to the error of ≥ 1 log_10_ units on a continuous LD_50_ scale.

* *p* < 0.05, TOPKAT model predictions versus all other models by using the permutation (10,000 times) test.

**Table 3:** Frequently used descriptors in a *k*NN Hybrid (THR = 15%) model.

Dragon chemical descriptor (label and occurrence)	Representation	T/N-T^a^	Example
nCH2RX (59%)	Alkyl halides	19/4	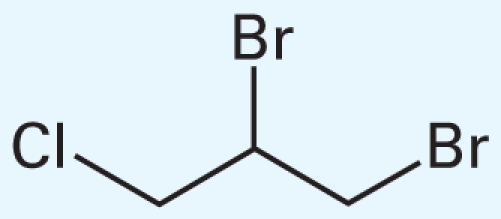 CAS no. 96-12-8
Br-091 (12%)			
Cl-086 (5%)			

B03[O-Cl] (55%)	Aryl halides, haloalkyl ethers	18/3	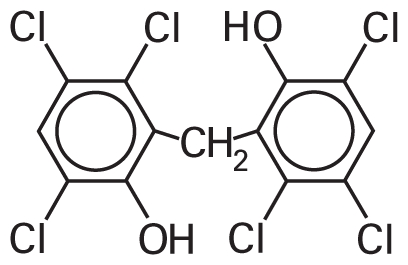 CAS no. 70-30-4
F03[O-Cl] (13%)			
B04[Cl-Cl] (7%)			
B05[O-Cl] (5%)			
B01[C-Br] (5%)			

nS (36%)	Thiophosphates	22/17	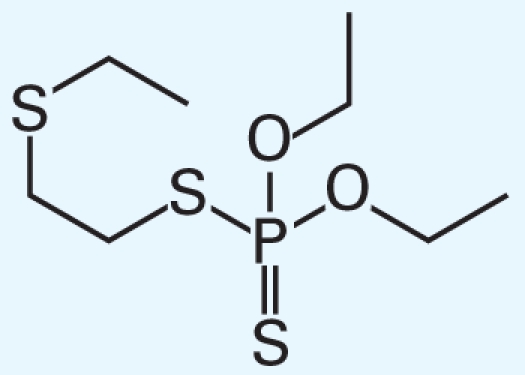 CAS no. 298-04-4
B04[C-S] (28%)			
B05[C-S] (26%)			
B03[C-S] (13%)			
B01[C-S] (12%)			
F05[C-S] (9%)			
F04[C-S] (7%)			
B02[C-S] (7%)			
B07[C-S] (4%)			

nRCN (21%)	Alkyl nitriles	5/1	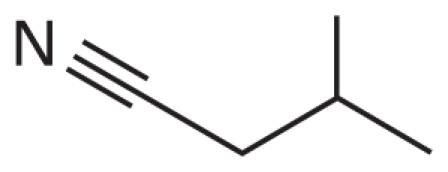 CAS no. 78-82-0
nTB (10%)			

C-001 (20%)	Methyl groups	25/29	CH_3_–[C,N,O,S,]...
C-005 (10%)			

Mv (38%)	Molecular size	—	
AMW (17%)			

F02[C-C] (13%)	Carbon backbone	—	

nCIC (10%)	Rings count	—	

ARR (8%), nCbH (9%), nCb- (5%)	Aromatic compounds	—	

a “T/N-T” is the number of “toxic” and “nontoxic” chemicals that represent the corresponding descriptor in the qHTS LD_50_ data set.

**Table 4:** Classifications for similar compounds.

Item no.	Compounds	qHTS profile^a^	Activity	Classification	Structure
1	X=Cl, Y=H; CAS no. 58-90-2	0000000111	1	1	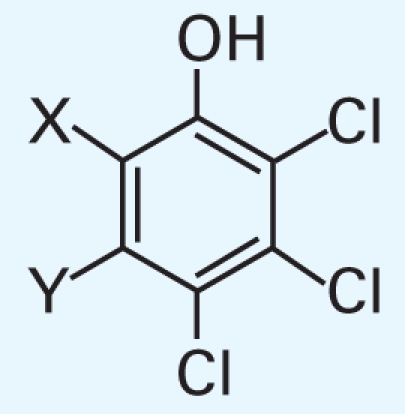
	X,Y=Cl; CAS no. 87-86-5	0000000111	1	1	
	X=H, Y=Cl; CAS no. 4901-51-3	0000001111	1	1	

2	X=H; CAS no. 71-41-0	0000000000	0	0	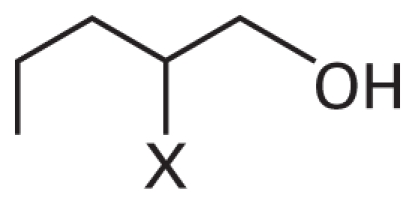
	X=CH_3_; CAS no. 105-30-6	0000000000	0	0	

3	X=H; CAS no. 141-78-6	0000000000	0	0	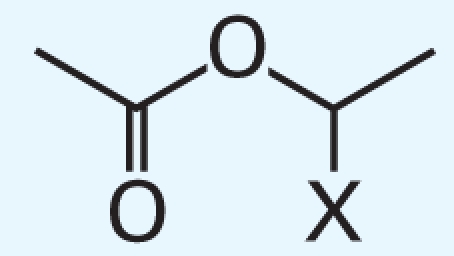
	X=CH_3_; CAS no. 108-21-4	0000000000	0	0	

4	X=H; CAS no. 100-52-7	0001010101	0	0	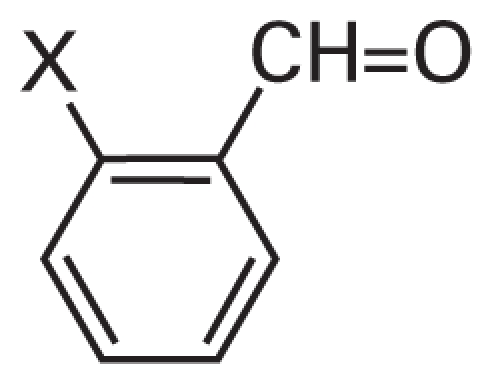
	X=CH_3_; CAS no. 529-20-4	0000000000	0	0	

5	CAS no. 74-96-4	0000000000	0	0.9	H–CH_2_–CH_2_–Br
	CAS no. 106-93-4	0000000001	1	0.9	Br–CH_2_–CH_2_–Br
	CAS no. 107-04-0	0000000000	1	1	Cl–CH_2_–CH_2_–Br

6	X=Me; CAS no. 7-50-58	0000000000	0	0.8	X—≡N
	X=Et; CAS no. 107-12-0	0000000000	1	0	
	X=i-Pr; CAS no. 78-82-0	0000000000	1	0	

7	X=1,3-di-Me-But, Y=H, Z=Ph; CAS no. 793-24-8	0000011111	0	0.6	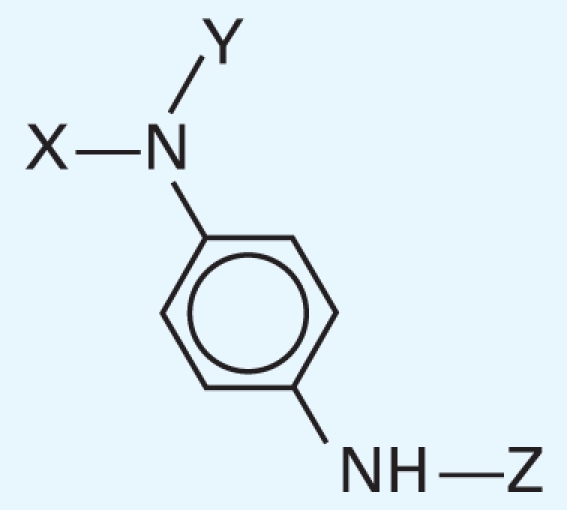
	X,Y=CH_3_, Z=H; CAS no. 99-98-9	0000011011	1	0.6	
	X=H, Y,Z=2-But; CAS no. 101-96-2	1011111111	1	0.7	

8	CAS no. 123-38-6	0000000000	0	0	CH_3_–CH_2_–CH=O
	CAS no. 107-02-8	0000000111	1	0.3	CH_2_=CH–CH=O

9	CAS no. 78-93-3	0000000000	0	0	CH_3_–CH_2_–C(CH_3_)=O
	CAS no. 78-94-4	0000000000	1	0	CH_2_=CH–C(CH_3_)=O
	CAS no. 78-92-2	0000000000	0	0	CH_3_–CH_2_–C(CH_3_)–OH

Abbreviations: But, butyl; Et, ethyl; i-Pr, isopropyl; Me, methyl; Ph, phenyl. Only bits of five highest concentrations are shown. “Activity,” experimental activity class; “Classification,” predicted class (average across all random forest and *k*NN models).

a A concentration–response curve fingerprint based on the five highest concentrations only (see “Materials and Methods”) derived at THR = 15%, MXDV = 5% (maximum across 13 cell lines).
